# Terpenoids Associated with the Resistance of Poplar to Canker Disease as Revealed by Transcriptomics and Metabolomics Analyses

**DOI:** 10.3390/biology15030226

**Published:** 2026-01-26

**Authors:** Jingping Dong, Jinxin Zhou, Ye Zheng, Honghua Su, Yaohai Li, Yang Ci, Quzhen Gesang, Zhaolin Ji, Bingzhang Li

**Affiliations:** 1 Tibet Academy of Forest Trees, Lhasa 851400, China; 2 College of Plant Protection, Yangzhou University, Yangzhou 225009, China; jpdong8@126.com (J.D.);; 3 Service Center of Xinghua Modern Agriculture Development, Xinghua 225700, China

**Keywords:** conjoint analysis, *Cytospora* spp., metabolome, poplar, transcriptome

## Abstract

Poplar (*Populus* spp.) has a short growth cycle and considerable economic value. Also, it is highly susceptible to various diseases. Of these, the canker disease has a significant impact on the health and productivity of poplar plants. This study aimed to explore the resistance mechanism of poplar against *Cytospora* spp. by transcriptomics and metabolomics sequencing analyses that were performed after 0, 48, and 96 h of inoculation in resistant and susceptible varieties of poplar. Both transcriptome and metabolome profiles demonstrated a robust activation of defense mechanisms in the resistant variety upon pathogen challenge. Of all differentially expressed genes (DEGs), 175 DEGs were selected and combined with 65 candidate differentially accumulated metabolites (DAMs) following the same trend for further conjoint analysis. The combined analysis results revealed a significant correlation between 8 DAMs and 72 DEGs. Sixteen DEGs associated with each DAM were screened to verify their expression levels using quantitative reverse transcription–polymerase chain reaction. Most of the eight DAMs belong to the category of terpenoids. It suggested that terpenoids were crucial components of a poplar’s defense response to canker disease. This study provides a genetic basis and novel insights for future investigations of poplar resistance mechanisms against *Cytospora* spp.

## Simple Summary

Canker disease, caused by *Cytospora* spp., is a major threat to health, and a major cause of productivity loss, in poplar. Through the combined analysis of transcriptomics and metabolomics sequencing, we screened 8 differentially accumulated metabolites that belong to terpenoids and 16 differentially expressed genes with highly quantitative fold change values after inoculation. It provides a genetic basis and novel insights for future investigations of poplar resistance mechanisms against *Cytospora* spp.

## 1. Introduction

Poplar (*Populus* spp.) is characterized by a wide range of suitable planting areas, adaptability, rapid growth, substantial woody biomass, and the industrial versatility of its timber. Therefore, it has become one of the main tree species for afforestation to cope with climate change worldwide [1,2]. Statistics reveal that the total area of poplar forests in China has significantly increased to 10.10 million hectares, and the cultivated area ranks first in the world [3]. Poplar is the dominant tree species in Tibet, accounting for 45.34% of the planted forests in the area. It is widely distributed, ranging from an altitude of 1600-4050 m. However, the health and productivity of these vast poplar resources are increasingly threatened by biotic stresses and associated diseases.

Among these, poplar canker disease, also known as bark rot, has a significant impact on the health and productivity of poplar [4]. The poplar canker disease is widespread in China, especially in the major planting areas of poplar in the northeast, north, and northwest regions of China [5]. Poplar canker disease is a latent disease caused by a weak parasitic pathogen. The harsh climatic conditions in Tibet, including low temperatures, drought stress, and extreme temperature fluctuations, can reduce tree vigor and aggravate the occurrence of canker disease. *Cytospora* spp. can only germinate specifically in response to polar-derived compounds exuded from poplar tissues, indicating that the pathogens involve a sophisticated chemotactic response. This host–pathogen specificity underscores the critical need to identify the key metabolites that either trigger infection or are produced as part of the poplar’s defense response.

Terpenoids are widespread in plants, fungi, bacteria, and some animals [6,7,8]. In plant–pathogen interactions, terpenoids represent a crucial class of defensive secondary metabolites. These compounds, built from isoprene units, are ubiquitous across the plant kingdom and are broadly categorized into primary metabolites (phytohormones, steroids, carotenoids, and polyterpene alcohols) and secondary metabolites (monoterpene menthol, sesquiterpene artemisinin, diterpene paclitaxel, triterpene ginsenoside, and tetraterpene carotenoids) [9]. Terpenoids participate in plant growth, development, and defense. The main functions of terpenoids in plants include enhancing plant disease resistance and helping plants to resist natural enemies in direct and indirect manners [10,11]. The linalool, whose pathogen-induced biosynthesis is catalyzed by the terpene synthase gene *Sobic.004G019400*, confers resistance against anthracnose both by directly disrupting fungal cell membrane integrity and energy metabolism and by potentiating host defense responses [12]. However, despite their recognized importance, the specific regulatory networks and the full spectrum of terpenoid-mediated defense mechanisms in poplar against canker disease remain poorly elucidated.

Currently, studies employing integrated transcriptomics and metabolomics to deconstruct the defense response of poplar to *Cytospora* spp. are scarce. While terpenoids are known defense compounds, their specific role in poplar resistance to canker disease remains elusive. This study was designed to fill this gap. We aimed to profile the dynamic changes in differentially expressed genes (DEGs) and secondary metabolites related to defense in the resistant and susceptible varieties at 0, 48, and 96 h post-inoculation with *Cytospora* spp. Through this work, we seek to delineate the key pathways and critical metabolites involved in the defense response, thereby uncovering novel molecular strategies for enhancing poplar resistance to canker disease.

## 2. Materials and Methods

### 2.1. Plant Materials and Treatments

Well-grown, uniform, pest-free plants of *Populus szechuanica* Schneid. Var. *tibetica* (as the susceptible material, called ‘S’ in the study), and *Populus tomentosa* Carr. (as the resistant material, called ‘R’ in the study), were selected as test varieties. These branches were from different trees in the same species. They were both planted in the Forest Science Research Institute of Tibet Autonomous Region. The purified pathogenic fungi (*Cytospora* spp.) were inoculated on potato dextrose agar culture medium and incubated at 25 °C in the dark for 2–3 days. The strain was obtained from the previous process of isolation, purification, verification, and preservation of our laboratory. The mycelium was inoculated onto branches that were wounded with a heated instrument at 180 °C for 10 s. After inoculation, the branches were placed in the incubator. Three biological replicates were employed for all treatments. The branches were sampled after 0, 48, and 96 h of inoculation, immediately frozen in liquid nitrogen, and stored at −80 °C for subsequent transcriptome and metabolome analyses by Biotree Co., Ltd. (Shanghai, China). The samples were named as the S0, S48, S96, R0, R48, and R96 groups.

### 2.2. Transcriptome Analysis

Total RNA was isolated using the Total RNA Extraction Reagent (Vazyme, Nanjing, China; Catalog No. R401-01). RNA degradation and contamination were detected using 1% agarose gel. RNA integrity was evaluated using the Agilent 5400 Fragment Analyze System (Agilent Technologies, Santa Clara, CA, USA). The first strand of cDNA was synthesized in the reverse transcriptase system with mRNA enriched with polyA, using oligo(dT) magnetic beads from total RNA as the template, six-base random oligonucleotides as primers, and dNTPs (uploaded as the ID no. PRJNA1333594) as raw materials. The second strand of cDNA was synthesized and purified using DNA polymerase I. The purified double-stranded cDNA was subjected to terminal repair, followed by A-tailing, and ligated with a sequencing adaptor. Then, double-stranded cDNA fragments (250–350 bp) were screened using Agencourt AMPure XP Beads (Beckman Coulter, Brea, CA, USA). The target double-stranded cDNA was subjected to polymerase chain reaction (PCR) amplification and purification. Finally, the library was obtained. The raw data were filtered to be clean data based on containing sequencing adapters; the average quality threshold was higher than Q20 using a 4-base sliding window, filtering out reads with more than 5 uncertain bases, deleting the reads that were shorter than 15 bases. The effective concentration (more than 2 nM) of the high-quality library was accurately quantified using quantitative reverse transcription–polymerase chain reaction (qRT-PCR).

The high-quality (clean reads) reads were compared with the reference genome of *P. trichocarpa* (BioProject: PRJNA10772 and PRJNA17973, accessed on 5 October 2022) using HISAT 2.0 to obtain the positional information on the reference genome or genes. Then, StringTie 3.00 was used for the quantitative analysis of the expression of all sample genes. The DESeq2 1.45.1 package was used to conduct the differential expression analysis between sample groups. Genes with an adjusted |log2 Fold Change| > 1.0 and *p* < 0.05 were considered as differentially expressed. The differentially expressed genes (DEGs) were compared between samples that were taken at 48 h and 96 h after inoculation and that taken at 0 h, and compared between two species. The screened DEGs were annotated against the Gene Ontology (GO), Kyoto Encyclopedia of Genes and Genomes (KEGG), Eukaryotic Orthologous Groups (KOGs), NCBI non-redundant (NR) protein sequences, SWISS-PROT Protein Knowledgebase (SWISS-PROT), Protein Families database (Pfam), and transcription factor databases to obtain information on protein functional annotation corresponding to all DEGs. All DEGs had their expression trend conducted using the software (https://www.omicshare.com/tools/home/report/reporttrend.html, accessed on 14 January 2026) with *p* value < 0.05.

### 2.3. Metabolome Analysis

All the samples per treatment were processed as follows. The branches were vacuum freeze-dried and then powdered in a grinder at 60 Hz for 30 s. Further, 25 ± 1 mg of sample powder was weighed, dissolved in 1000 μL of extraction solution (methanol: acetonitrile: water = 2:2:1, *v*/*v*/*v*), vortexed for 4 min, sonicated for 5 min, and centrifuged at 12,000× *g* for 15 min. The supernatant was pooled, filtered through a 0.22 μm microporous membrane, and stored in a Petri dish with equal amounts for subsequent LC-MS analysis.

The analysis was conducted using a quadrupole time-of-flight mass spectrometer (Thermo Fisher Scientific, Waltham, MA, USA) with a 2.1 × 50 mm^2^, 2.6-μm Phenomenex Kinetex C18 column. In both ESI-positive and ESI-negative modes, the mobile phase comprised the following: A = 0.01% acetic acid, B = isopropanol: acetonitrile (1:1, *v*/*v*). The primary and secondary mass spectrometry data were collected using the Orbitrap Exploris 120 (Xcalibur 2.0; Thermo Fisher Scientific, Waltham, MA, USA). Detailed parameters were as follows: sheath gas flow rate, 50 Arb; auxiliary gas flow rate, 15 Arb; capillary temperature, 320 °C; full MS resolution, 60,000; MS/MS resolution, 15,000; collision energy, Stepped Normalized Collision Energy (SNCE) 20/30/40; and spray voltage: 3.8 kV (positive) and −3.4 kV (negative).

Based on the self-built library (Biotree DB 3.0 and BT-Plant 1.1), ProteoWizard 3.0.24054 was used to process the MS data to convert to mzXML format. The data management was executed, mainly including deviation value filtering, missing value filtering, missing value recoding, and normalization. The principal component analysis (PCA) and Hierarchical Cluster Analysis (HCA) were performed using SIMCA 18.0.1 (Sartorius Stedim Data Analytics AB, Umea, Sweden). Orthogonal partial least squares discriminant analysis (OPLS-DA) was performed to assess the reliability of the application model. Based on OPLS-DA results, differentially accumulated metabolites (DAMs) were screened under the thresholds of variable importance in projection (VIP) higher than 1.0 and *p* value less than 0.05, combining VIP, |log2 Fold Change|, and *p* value. The DAMs were annotated, classified, and enriched employing the KEGG database. Likewise, the DAMs were compared between samples that were taken at 48 h and 96 h after inoculation and that taken at 0 h. To know the changing trend of the relative content of metabolites in different groups, the relative contents of all DAMs identified according to the screening criteria in all group comparisons were standardized by z-score, and then K-means cluster analysis was conducted.

### 2.4. Association Analysis of Transcriptome and Metabolome

A preliminary screening of transcriptome and metabolome data was conducted based on |log2 Fold Change| > 2.0 and |log2 Fold Change| > 1.0, respectively. In the transcriptome, the genes upregulated in both *P. szechuanica* and *P. tomentosa* after induction by the pathogen were selected. In the metabolome, the DAMs positively regulated only in the resistant variety were selected.

The complex relationship between genes and phenotypes makes it difficult to determine the key signaling pathways. Also, individual omics studies often fail to achieve the intended research goals. Therefore, a multi-omics combined analysis was employed in this study. The aggregation of samples within each group and the overall distribution trend of each sample were analyzed using PCA and IPCA based on the DEGs and DAMs. Then, different omics findings were compared to examine the patterns in the similarities and differences in the sample distribution trends, if any.

### 2.5. Gene Expression Verification Using qRT-PCR

A total of 16 candidate DEGs were subjected to qRT-PCR to validate the DEGs detected by RNA-seq sequencing. The primers were designed using Primer 5.0 and synthesized by Sangon Biotech (Shanghai, China). These primers are listed in Table S2. qRT-PCR was performed on a CFX96 Touch Real-Time PCR Detection System (Bio-Rad, Hercules, CA, USA) using the ChamQ Universal SYBR qPCR Master Mix (Vazyme, Nanjing, China; Catalog No.: Q411-02). Three biological replicates and three technical replicates per treatment were used with *P. trichocarpa* and *P. szechuanica*. Actin was used as the internal reference gene (*PtActin*) [13]. The 2^−ΔΔCt^ method was used for determining the gene expression ratios [14].

## 3. Results

### 3.1. Transcriptomic Profiling Reveals a Strong Defense Response in the Resistant Cultivar

#### 3.1.1. Quality Control Analysis and Transcriptome Data Description

The raw reads of each sample ranged from 37,012,026 to 69,733,872. Then, the clean reads were generated from 36,726,152 to 69,359,674 after quality filtering. The clean bases of each sample were distributed in the range of 5.536–10.435 G. Q20 was more than 99.36%, Q30 was more than 97.02%, and the GC content was from 43.26 to 44.60. The reads uniquely mapped were in the range of 89.09% to 97.34%. These results indicated that the quality of the transcriptome sequencing data was extremely high and reliable (Table S1).

PCA was used to determine the reliability among different biological replicates. The findings are shown in Figure S1. The three replicates within the group were close to each other, indicating good repeatability of each sample. A clear separation trend was observed between groups, with PC1 accounting for 35.6% and PC2 accounting for 25.3% of the variance.

#### 3.1.2. Screening, Identification, and Analysis of DEGs

**Figure 1:**
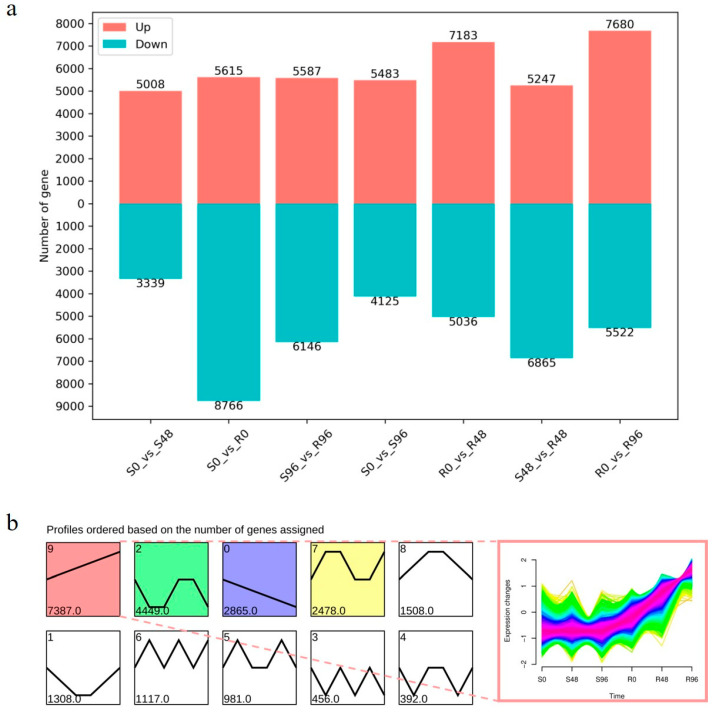
Analysis of DEGs. (**a**) Bar chart of the number of DEGs in each comparison group. (**b**) Trend analysis of all DEGs.

The bar chart showing the number of DEGs in the comparison groups was created using |log^2^ Fold Change| > 1.0 and *p* value < 0.05 as the screening criteria, as shown in Figure 1a. A total of 12,219 and 13,202 DEGs were identified after 48 and 96 h of inoculation, respectively, compared with 0 h of inoculation in the ‘R’ cultivar. Further, 8347 and 9608 DEGs were identified after 48 and 96 h of inoculation, respectively, compared with 0 h of inoculation in the ‘S’ cultivar. Also, 14,381, 12,112, and 11,733 DEGs were identified after 0, 48, and 96 h of inoculation, respectively, in the ‘R’ cultivar compared with the ‘S’ cultivar. Moreover, more upregulated DEGs were identified after 48 and 96 h of inoculation in the ‘R’ cultivar compared with the ‘S’ cultivar.

**Figure 2:**
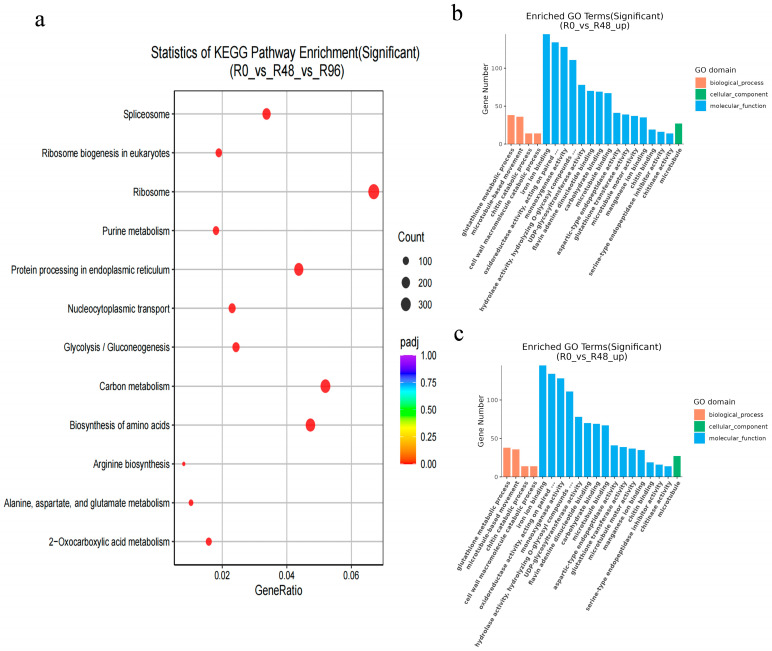
Analysis of DEGs. (**a**) KEGG enrichment analysis of DEGs in R0 versus R48 versus R96. The image was drawn on an online software (https://www.omicshare.com/tools/Home/Soft/trend, accessed on 14 January 2026); (**b**,**c**) GO annotation classifications of upregulated DEGs in R0 versus R48, and R0 versus R96.

GO and KEGG enrichment analyses were performed on DEGs in the ‘R’ cultivar induced by pathogenic infection to determine the functional categories and distribution patterns of resistance genes. In the GO enrichment analysis, the DEGs with the highest abundance belonged to the molecular function category related to oxidoreductase activity. The KEGG database was used to identify pathways showing significant changes, with ‘Ribosome,’ ‘Carbon metabolism,’ and ‘Biosynthesis of amino acids’ being representative (Figure 2a–c). The findings showed that oxidative enzymes, amino acids, and carbon metabolism played important roles in resisting pathogen infection in *Populus*.

An expression trend distribution analysis was conducted to understand and screen for resistance DEGs. A total of 7387 DEGs were upregulated, with expression levels higher in the ‘R’ cultivar compared with the ‘S’ cultivar. The number of DEGs following this trend was the highest. Further, 2865 DEGs were downregulated, with expression levels lower in the ‘R’ cultivar compared with the ‘S’ cultivar (Figure 1b). Of 7387 upregulated DEGs, 552 were screened based on |log^2^ Fold Change| > 2.0. These DEGs were upregulated after 48 and 96 h of inoculation in the ‘R’ cultivar, but were not expressed after 0 h of inoculation. The DEGs were excluded based on Fragments Per Kilobase of Exon Per Million Mapped Reads (FPKM) value < 10.0. Finally, 175 DEGs remained for the subsequent analysis.

### 3.2. Metabolomic Profiling Identifies Key DAMs Associated with Disease Resistance

**Figure 3:**
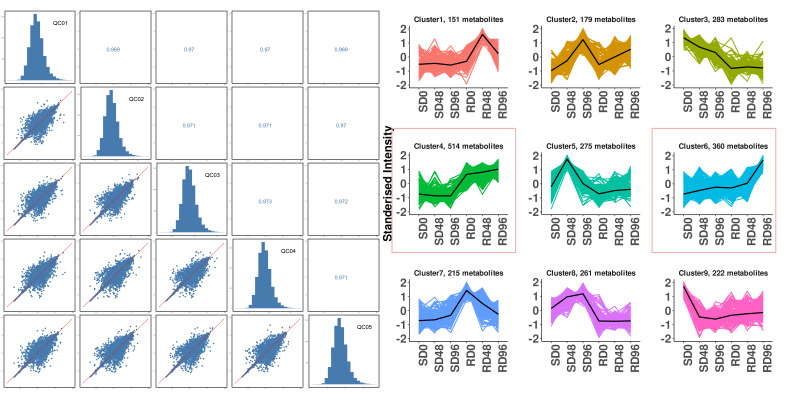
Analysis of DAMs. (**a**) Analysis of QC samples. (**b**) Principal component analysis of all samples.

Untargeted metabolomics sequencing was performed on 36 samples, and quality control analysis was performed on the sequencing data to investigate the metabolic differences between *P. szechuanica* and *P. tomentosa* after inoculation. The correlation coefficients exceeding 0.90 in the analysis of all the quality control (QC) samples were highly consistent (Figure 3a). In PCA, the six replicates within the group were close to each other, indicating good repeatability of each sample, as shown in Figure S2b. A clear separation trend was noted between groups, with PC1 determining 56.8% and PC2 determining 9.7% of the variation rate, respectively.

A total of 2541 metabolites were identified in the 36 samples and categorized into 8 superclasses, 59 classes, and 256 subclasses (Figure S2c). K-means clustering analysis was used to determine the changing trends of the DAMs in different groups. It revealed nine major clusters with the same changing trend. Further, 514 (Cluster 4) and 360 DAMs (Cluster 6) had a higher relative content in the ‘R’ cultivar compared with the ‘S’ cultivar. All of them exhibited a positive trend in both ‘R’ and ‘S’ cultivars after inoculation. Among 874 DAMs (Cluster 4 and Cluster 6), 65 positively regulated only in the ‘R’ cultivar were selected for subsequent integrated analysis (Figure 3b).

### 3.3. Correlation of Transcriptome and Metabolome Data Reveals Hub Metabolites Contribution to Disease Resistance in Poplar

**Figure 4:**
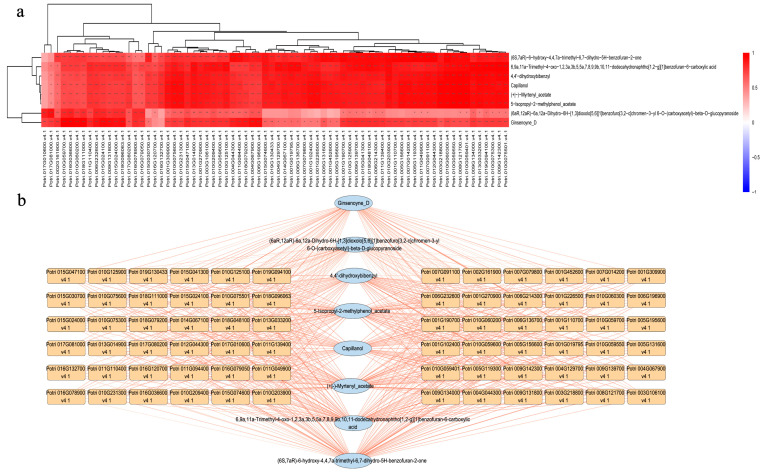
Correlation analysis of candidate DEGs and DAMs. (**a**) Correlation heatmap of DEGs and DAMs. The darker the shade of red, the higher the correlation coefficient. * indicates *p* value < 0.05; ** indicates *p* value < 0.01; *** indicates *p* value < 0.001. (**b**) Correlation network of candidate DEGs and DAMs by Cytoscape version 3.10.3.

**Figure 5:**
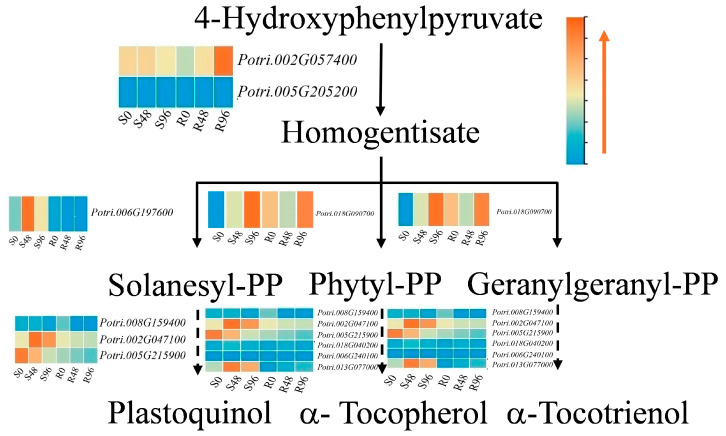
DEGs and DAMs related to biosynthetic pathways of terpenoids in poplar after inoculation. Orange font denotes significantly increased expression levels. S0, S48, S96 indicate the FPKM values of sampling time points after inoculation from sensitive poplar. R0, R48, R96 indicate the FPKM values of sampling time points after inoculation from resistant poplar. The color scale from blue to orange indicates low to high expression levels.

The integrated analysis of transcriptomics and metabolomics data was performed to know a deeper understanding of the molecular basis of life activities, the revelation of disease mechanisms, and the guidance of biotechnological applications, and to provide a more comprehensive and accurate research perspective. The result showed that eight DAMs [(+|−)-myrtenyl acetate, (6aR, 12aR)-6a, 12a-dihydro-6H-[1, 3] dioxolo [5, 6] [1] benzofuro [3, 2-c]chromen-3-yl 6-O-(carboxyacetyl)-beta-D-glucopyranoside, (6*S*, 7aR)-6-hydroxy-4, 4, 7a-trimethyl-6, 7-dihydro-5H-benzofuran-2-one, 4, 4′-dihydroxybibenzyl, 5-isopropyl-2-methylphenol acetate, 6, 9a, 11a-trimethyl-4-oxo-1, 2, 3a, 3b, 5, 5a, 7, 8, 9, 9b, 10, 11-dodecahydronaphtho [1, 2-] [1] benzofuran-6-carboxylic acid, capillanol, and ginsenoyne_D] were correlated with almost all candidate DEGs (Figure S1). After excluding the DEGs with a low FPKM value, 72 DEGs were selected for further conjoint analysis with 8 DAMs (Figure 4a). Four DAMs were terpenoids, two were shikimates and phenylpropanoids, and one was a fatty acid. (6aR, 12aR)-6a, 12a-Dihydro-6H-[1, 3] dioxolo [5, 6] [1] benzofuro [3, 2-c] chromen-3-yl 6-O-(carboxyacetyl)-beta-D-glucopyranoside was associated with the largest 69 DEGs. (+|−)-Myrtenyl acetate was associated with the lowest 23 DEGs (Figure 4b); the last capillanol had not successfully enriched into a certain superclass. All terpene synthesis pathways identified in the transcriptomics and metabolomics analyses were compiled, and the expression levels of the DEGs in each pathway were included (Figure 5).

### 3.4. Filtering of Candidate DEGs

**Figure 6:**
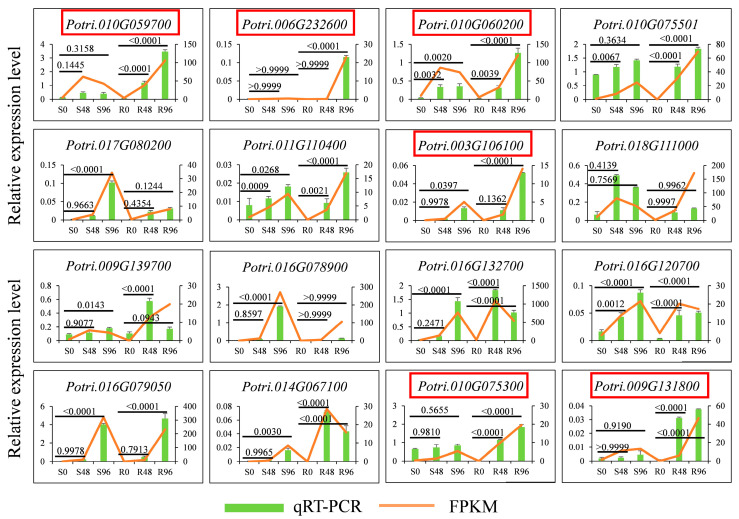
Expression profiles of candidate DEGs. The green bar chart shows the relative expression levels of DEGs detected using qRT-PCR. The orange line represents the FPKM values of DEGs obtained from transcriptomics analysis. Genes outlined in red represent candidate genes.

A total of 16 DEGs correlated with all eight candidate DAMs were screened for their expression levels using qRT-PCR to further verify the reliability of the transcriptome sequencing. As shown in Figure 6, the expression trends from the qRT-PCR results were consistent with the FPKM values from the transcriptome analysis, suggesting that the data were reliable and suitable for the aforementioned analyses. The qRT-PCR results showed that all DEGs had high quantitative fold change values with positive regulation after inoculation. The expression levels of most DEGs were higher in the ‘R’ cultivar compared with the ‘S’ cultivar, except for *Potri.017G080200*, *Potri.018G111000*, and *Potri.016G120700*. The functions of six DEGs were hypothetical; these included *Potri.016G79050*, *Potri.016G78900*, *Potri.009G131800*, *Potri.011G110400*, *Potri.010G75501*, and *Potri.010G75300*.

The convergent expression patterns of *Potri. 010G059700*, *Potri. 006G232600*, *Potri. 010G060200*, *Potri. 003G106100*, *Potri. 010G075300*, and *Potri. 009G131800* prompted their selection as the focal genetic basis for our subsequent research (Figure 6).

## 4. Discussion

Poplar (*Populus* spp.), a species of considerable ecological and ornamental importance in China, faces persistent threats from canker disease caused by *Cytospora* spp. Despite its widespread cultivation, the molecular mechanisms underlying poplar’s defense response against this pathogen remain insufficiently characterized. In this study, we employed an integrated transcriptomics and metabolomics sequencing analyses to compare resistant (‘R’) and susceptible (‘S’) poplar cultivars following inoculation *Cytospora* spp. at different time points. Our analysis focused on identifying differentially expressed genes (DEGs) that were induced by pathogen challenge to consistently expressed high levels in the ‘R’ cultivar, thereby pinpointing candidate genes associated with disease resistance.

In the transcriptome, a KEGG pathway analysis was conducted on DEGs showing upregulated expression across the S0, S48, S96, R0, R48, and R96 groups. ‘Ribosome,’ ‘Carbon metabolism,’ and ‘Biosynthesis of amino acids’ were representative in the KEGG analysis results. Ribosome biosynthesis is integral to be modulated in response to external stimuli, including biotic and abiotic stresses, enabling plants to reallocate resources toward defense-related protein synthesis [15,16]. Additionally, pathogens often manipulate host carbon metabolism to acquire nutrients such as glucose, suggesting that poplar may restructure its primary metabolism to limit nutrient availability to the fungus [17,18]. Similarly, amino acid biosynthesis pathways have been implicated in plant-pathogen interactions, with previous studies demonstrating that silencing specific amino acid biosynthesis genes in hosts can enhance resistance against oomycete pathogens [19]. These findings collectively indicate that poplar reconstitutes its primary metabolic processes as part of a coordinated defense strategy against *Cytospora* spp. infection.

Beyond primary metabolism, secondary metabolites play extremely important roles in plant defense against various stresses. In this study, a total of 2541 metabolites were identified and categorized into 8 superclasses, 59 classes, and 256 subclasses. The overall increase in abundance of differentially accumulated metabolites (DAMs) suggests a systemic activation of secondary metabolic pathways in response to pathogen challenge. Among these, 65 DAMs were significantly more abundant in the ‘R’ cultivar than in the ‘S’ cultivar, supporting their potential role in resistance mechanisms. Integrated pathway analysis further highlighted the enrichment of terpenoid metabolism, a class of compounds widely recognized for their antimicrobial and signaling functions in plant defense.

The correlation-based combined analysis of the transcriptome and metabolome in poplar confirmed that the metabolic pathways of terpenoids were relatively enriched. We identified 16 DEGs significantly associated with eight candidate resistance-related metabolites. Among these, 13 DEGs showed higher expression in the ‘R’ cultivar, reinforcing their likely role in modulating poplar resistance. The remaining DEGs included nucleoredoxin (NRX), aspartyl protease family protein, cationic peroxidase, acetylserotonin O-methyltransferase, nudix hydrolase, and pectinesterase 2. NRXs contain three tandemly arranged thioredoxin-like (TRX-like) modules and are localized to both the nucleus and cytoplasm [20]. *AtNRX1* can induce disease resistance in plants as a molecular sensor through its redox-dependent structural modification [21]. In our study, two NRX genes (*Potri.010G060200* (nucleoredoxin 1) and *Potri.010G59700* (nucleoredoxin 1)) were both upregulated and were at higher levels in the ‘R’ cultivar than in the ‘S’ cultivar. Similarly, cationic peroxidase-previously linked to disease resistance in barley and maize-may contribute to cell wall strengthening or oxidative burst in poplar [22]. Nudix hydrolase modulates the levels of the substrates to maintain physiological homeostasis as a ‘housekeeping’ enzyme. *AtNUDT7* has been identified as a negative regulator of the defense response in *Arabidopsis* [23]. However, the expression of *Potri.003G106100* (nudix hydrolase 2) was higher in the ‘R’ cultivar, indicating species-specific regulatory roles that merit further functional validation. The vast chemical diversity of terpenoids, stemming from the modular assembly and cyclization of isoprene units into distinct classes (e.g., monoterpenes, sesquiterpenes, diterpenes, and triterpenoids), directly underpins their broad spectrum of biological activities and functional specificity [24]. This study not only elucidates the crucial mechanism of terpenoids in poplar canker resistance, but, more importantly, it pinpoints the core genes and metabolites governing this process. These findings provide clear molecular targets for poplar resistance breeding, enabling precise and efficient genetic improvement through marker-assisted selection or gene-editing technologies. Building on these findings, our next step is to further characterize the specific antimicrobial compounds identified in this study and elucidate how their structural properties influence the bioactivity of the product, completing a full circle from inquiry to application.

## 5. Conclusions

To further study the resistance mechanism of poplar to canker disease, in this study, metabolome and transcriptome sequencing was performed at different times after inoculation (S0, S48, S96, R0, R48, R96). By the trend analysis of transcriptome sequencing and untargeted LC-MS metabolomics and twice combined analysis, we screened the last candidate eight metabolites in the untargeted LC-MS metabolomics results, most of them belonging to terpenoids. There were 16 DEGs associated with each metabolite. All the candidate DEGs were highly expressed after inoculation. So, terpenoids can induce the expression of resistance genes to enhance the poplar’s resistance.

## Data Availability

All data generated or analyzed during this study are included in this published article.

## Supplementary Materials

The following supporting information can be downloaded at https://www.mdpi.com/article/10.3390/biology15030226/s1, Figure S1. Principal component analysis of all groups; Figure S2. Correlation analysis of primary screened DEGs and DAMs; Table S1. Data quality assessment of every sample; Table S2. Primers and annotations of candidate DEGs for qRT-PCR.

## Abbreviations

The following abbreviations are used in this manuscript:

DEGs	Differentially Expressed Genes
PCR	Polymerase Chain Reaction
qRT-PCR	Quantitative Reverse Transcription–Polymerase Chain Reaction
GO	Gene Ontology
KEGG	Kyoto Encyclopedia of Genes and Genomes
KOG	Eukaryotic Orthologous Groups
NR	NCBI Non-Redundant Protein Sequences
SWISS-PROT	SWISS-PROT Protein Knowledgebase
Pfam	Protein Families Database
SNCE	Stepped Normalized Collision Energy
PCA	Principal Component Analysis
HCA	Hierarchical Cluster Analysis
OPLS-DA	Orthogonal Partial Least Squares Discriminant Analysis
DAMs	Differentially Accumulated Metabolites
VIP	Variable Importance in Projection
QC	Quality Control
NRX	Nucleoredoxin
