# Supplementary figures and images for: Terpenoids Associated with the Resistance of Poplar to Canker Disease as Revealed by Transcriptomics and Metabolomics Analyses
Source: Biology (Basel). 2026 Jan 26;15(3):226. doi: 10.3390/biology15030226 (PMC12897179; doi:10.3390/biology15030226)

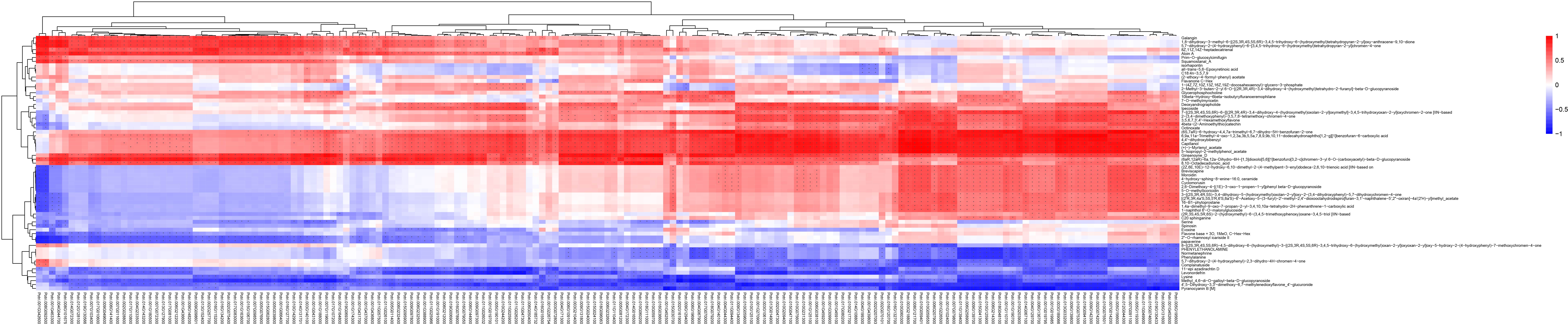

Supplement: Supplementary file 1 [file biology-15-00226-s001.zip › Figure S1.jpg]

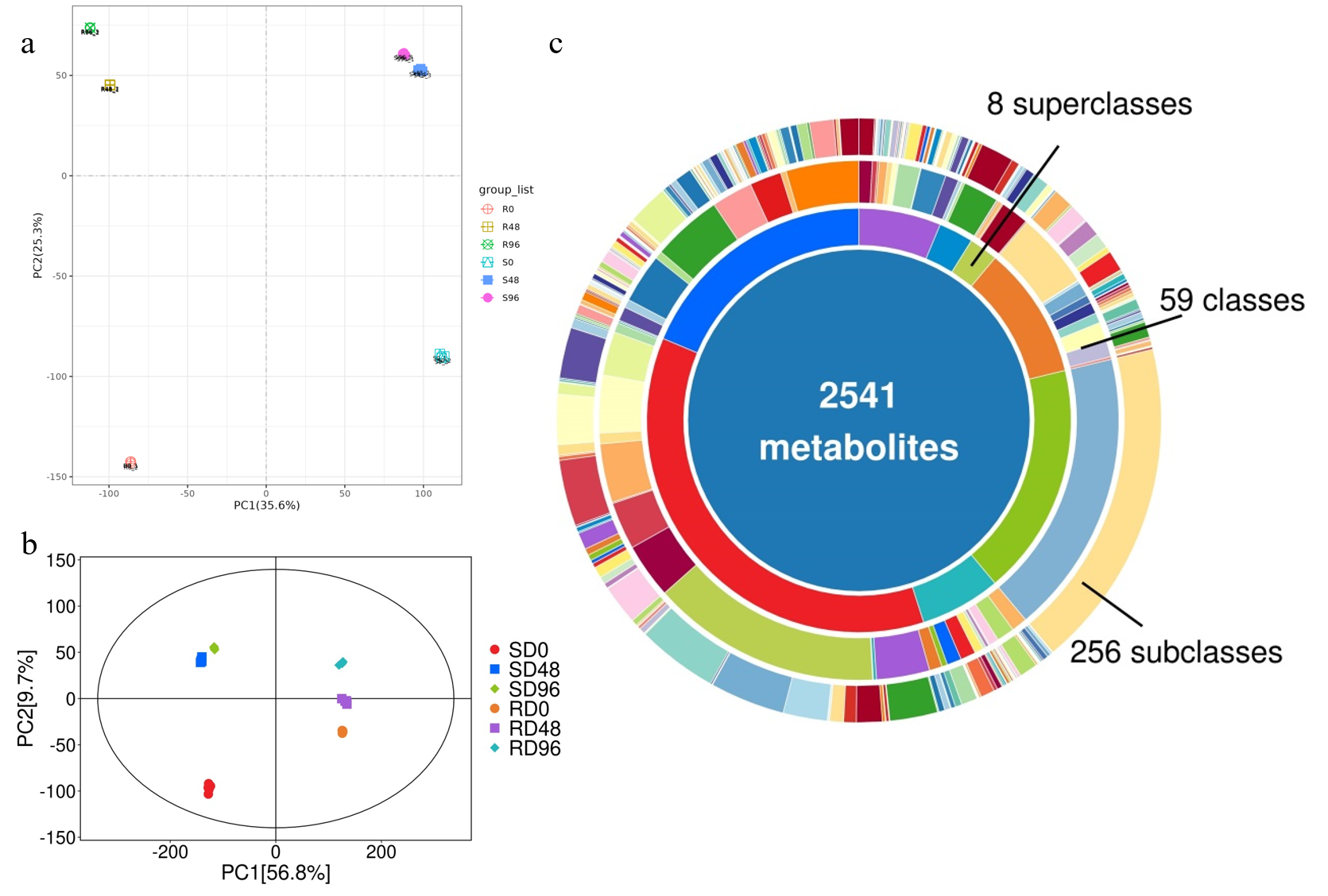

Supplement: Supplementary file 1 [file biology-15-00226-s001.zip › Figure S2.png]
